# What factors determine Belgian general practitioners’ approaches to detecting and managing substance abuse? A qualitative study based on the I-Change Model

**DOI:** 10.1186/1471-2296-15-119

**Published:** 2014-06-14

**Authors:** Frederic Ketterer, Linda Symons, Marie-Claire Lambrechts, Philippe Mairiaux, Lode Godderis, Lieve Peremans, Roy Remmen, Marc Vanmeerbeek

**Affiliations:** 1 Department of General Practice/Family Medicine, University of Liege, Avenue de l’Hôpital 3, CHU B23, Liege 4000, Belgium; 2 Department of Primary and Interdisciplinary Care, University of Antwerp, Campus Drie Eiken, R3, Universiteitsplein 1, Wilrijk 2610, Belgium; 3 KU Leuven, University of Leuven, Centre for Environment and Health, Kapucijnenvoer 35/5, blok D – box 7001, Leuven 3000, Belgium; 4 Department of Occupational Health and Health Promotion, University of Liege, Avenue de l’Hôpital 3, CHU B23, Liege 4000, Belgium; 5 IDEWE, External Service for Prevention and Protection at Work, Interleuvenlaan 58, Heverlee 3001, Belgium; 6 Department of Public Health, Vrije Universiteit Brussels, Laarbeeklaan 103, Brussel 1090, Belgium

**Keywords:** General practitioners, Substance abuse, Attitudes of health personnel, Motivation, I-Change Model

## Abstract

**Background:**

General practitioners (GPs) are considered to play a major role in detecting and managing substance abuse. However, little is known about how or why they decide to manage it. This study investigated the factors that influence GP behaviours with regard to the abuse of alcohol, illegal drugs, hypnotics, and tranquilisers among working Belgians.

**Methods:**

Twenty Belgian GPs were interviewed. De Vries’ Integrated Change Model was used to guide the interviews and qualitative data analyses.

**Results:**

GPs perceived higher levels of substance abuse in urban locations and among lower socioeconomic groups. Guidelines, if they existed, were primarily used in Flanders. Specific training was unevenly applied but considered useful. GPs who accepted abuse management cited strong interpersonal skills and available multidisciplinary networks as facilitators.

GPs relied on their clinical common sense to detect abuse or initiate management. Specific patients’ situations and their social, psychological, or professional dysfunctions were cited as cues to action.

GPs were strongly influenced by their personal representations of abuse, which included the balance between their professional responsibilities toward their patients and the patients’ responsibilities in managing their own health as well the GPs’ abilities to cope with unsatisfying patient outcomes without reaching professional exhaustion. GPs perceived substance abuse along a continuum ranging from a chronic disease (whose management was part of their responsibility) to a moral failing of untrustworthy people. Alcohol and cannabis were more socially acceptable than other drugs. Personal experiences of emotional burdens (including those regarding substance abuse) increased feelings of empathy or rejection toward patients.

Multidisciplinary practices and professional experiences were cited as important factors with regard to engaging GPs in substance abuse management. Time constraints and personal investments were cited as important barriers.

Satisfaction with treatment was rare.

**Conclusions:**

Motivational factors, including subjective beliefs not supported by the literature, were central in deciding whether to manage cases of substance abuse. A lack of theoretical knowledge and training were secondary to personal attitudes and motivation. Personal development, emotional health, self-awareness, and self-care should be taught to and fostered among GPs to help them maintain a patient-centred focus. Health authorities should support collaborative care.

## Background

The social and economic effects of alcohol and other drugs on society are substantial, but they largely depend on the type of drug. In 2010, alcohol use was the third leading risk factor for global disease burden [1]. Alcohol use plays a role in more than 60 major diseases and injuries. Worldwide, it results in approximately 2.5 million deaths each year [2]. Occasional or regular heavy drinking can damage health [3]. In addition, the use of illicit drugs is an important and increasing contributor to the global burden of disease [1,4]. The United Nations Office on Drugs and Crime (UNODC) estimates that between 102,000 and 247,000 drug-related deaths occurred in 2011 [5]. Cannabis is the most frequently used illegal substance in Europe [6]. Benzodiazepine abuse is a problem that remains largely unrecognised in many countries [7]. Europe has the highest average consumption of sedative-hypnotics and anxiolytics [7].

In Belgium, 10% of alcohol consumers aged 15 or older are problematic drinkers [3]. In 2008, 15% of Belgians reported having used painkillers, tranquilisers, or sleeping aids over the past two weeks. Over the past 12 months, 5% and 1.5% of the population had used cannabis and another illegal drug (e.g., MDMA, cocaine, and heroin), respectively [3].

General practitioners (GPs) are considered to play a major role in detecting and managing the problems related to substance abuse, regardless of its legality. However, previous work by Glanz, Gabbay and Deehan in the United Kingdom demonstrated that GPs view alcohol or drug misusers as undesirable patients [8-11]. Difficulty in managing and treating these patients raises concerns about the GPs’ feeling of competence and their confidence [12]. Attempts to provide specific training on this topic by Strang and McCambridge showed a limited impact, particularly regarding motivational aspects; thus, a better understanding of GP views and perspectives on substance misuse and misusers is essential [13-15]. In Belgium, little is known concerning GPs’ interests and attitudes toward caring for these patients or their management skills with regard to substance abuse behaviour.

This study is part of the “Up to Date” research project seeking to describe the approaches of GPs and occupational physicians (OPs) to the detection and management of the abuse of alcohol, illegal drugs, hypnotics, and tranquilisers among the Belgian population and to recommend ways to promote multidisciplinary collaborative care for these patients [16]. This paper describes only the GP arm of the study; the symmetry between the GP and the OP arms limited the topic to the working Belgian population (18-65 years old).

## Methods

### Conceptual model

This qualitative survey sought to answer the following question: “What are the experiences, attitudes, perspectives, and decision-making skills of GPs with regard to the abuse of alcohol, illegal drugs, hypnotics, and tranquilisers?” The survey sought to understand GPs’ points of view. The representations of substance abuse were considered a “guide to action” [17,18]; thus, GPs’ opinions were used to understand how they act.

**Figure 1:**
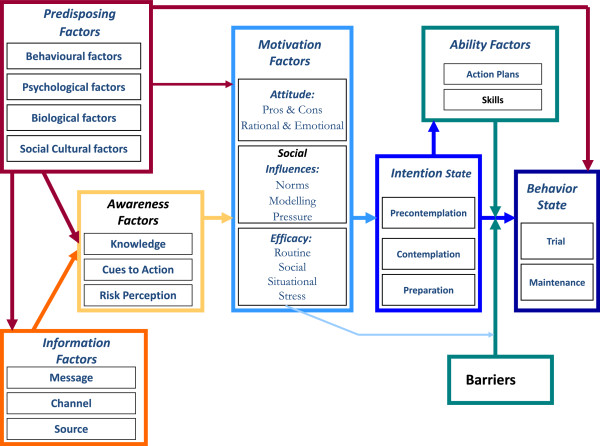
**The I-Change Model**[19]**.**

We used de Vries’ model as a conceptual framework (Figure 1) [19]. The Integrated Model (I-Change Model) for explaining motivational and behavioural change was derived from the Attitude–Social influence–Self-Efficacy Model [20,21], which is an integration of Ajzen’s Theory of Planned Behaviour, Bandura’s Social Cognitive Theory, Prochaska’s Transtheoretical Model, the Health Belief Model, and goal setting theories [22]. The I-Change Model was used to study various and complex clinical situations in patients and the behaviour of health professionals (smoking cessation, public perceptions regarding hereditary cancer, reporting of child abuse, and midwife behaviour) [19,23-25]. This broad applicability and the embedded motivational cycle guided our choice of this model.

Working with the I-Change model allowed us to distinguish between the factors that underlie GP decisions to care for patients with a problematic alcohol or drug use (e.g., knowledge and critical beliefs) and the factors resulting in differences between intentions and behaviours (e.g., skills). The major obstacles can be identified by deconstructing the process of intention into separate units (predisposal, awareness, information and motivational factors, abilities and barriers) and searching for the links between them. This article presents the results as a synthesis of the main results, and the results are classified according to the I-Change Model units.

### Data collection

#### Sampling procedure

Chairs of the local “GP Circles” and “Local Quality Evaluation Group” of the provinces of Antwerp and Liege connected us with GPs. These recruited GPs did not necessarily possess particular expertise in substance abuse; on the contrary, GPs working in specialised abuse clinics were excluded. First, the GPs were invited to complete a short questionnaire regarding their experience in the field and their practice profile. Second, the respondents were sampled to retrieve a variety of clinical profiles based on sex, age, reported experience in substance abuse management, practice location (rural or urban), and type of practice (single or group).

Ten GPs working in the Dutch-speaking province of Antwerp and ten working in the French-speaking province of Liege were selected. Their sociodemographic characteristics are summarised in Table 1.

**Table 1:** Sociodemographic characteristics of participants

	**Sex**		**Practice type**			**Experience**			**Practice location**	
	**M**	**F**	**Individual**	**Group**	**Medical homes**	**< 10 years**	**10 - 30 years**	**> 30 years**	**Urban**	**Rural**
Liege (French)	6	4	5	2	3	2	5	3	5	5
Antwerp (Dutch)	4	6	2	6	2	3	5	2	6	4
Total	10	10	7	8	5	5	10	5	11	9

In Belgium, GPs work in the context of a liberal healthcare system. The fee-for-service payment system predominates. However, GPs working in one of the 100 multidisciplinary primary healthcare centres (i.e., “medical homes”) are paid on a capitation basis. These centres, whose patients are bound by a contract of care, serve 3% of the population, primarily in urban and deprived areas [26].

### Interviewing procedure

Trained interviewers (FK, LS, and MV) conducted the dialogues at the GPs’ practices in the second half of 2012. A semi-structured interview guide, initiated from a clinical case, based on the I-Change Model and created via consensus between the researchers, was used (Additional file 1).

The duration of the interviews was approximately 1.5 hours. All were audio recorded and transcribed with the informed consent of the respondents. Data saturation was not examined because this study was an exploratory first step for creating a questionnaire.

### Analyses

The constant comparison technique was used in this analysis, which originates from the respondents’ replies verbatim. French- and Dutch-speaking researchers coded the first interviews independently using NVivo 10 software. The codebooks were then compared, discussed, and merged using an iterative consensus process in which the two teams approximated the wording of participants. The I-Change Model was used as a “sensitising concept” [27]. The codebooks were flexible until the end of the process. Both teams included bilingual researchers.

## Results

### Predisposing factors: the influence of practice location

Practice location was perceived as a strong influence on GPs’ experience with substance abuse management: Urban locations, patients of low socioeconomic class, and a high proportion of migrants were associated with a higher perceived prevalence of abuse, especially illegal drugs.

“In general, all goes well because we remain… [in] the privileged countryside; there are very, very few (and I am not a racist) foreigners. I do not have a single drug addict among my patients. There are no secrets; we deal with people who are clever and live in satisfactory socioeconomic conditions”. GP 16, Female (F), 35 year (y), French-speaking (Fr)

Abuse was mentioned among patients with low socioeconomic level, young age, psychiatric problems, social and professional dysfunctions, private life problems, social and ethnic origin (migrants from northern Africa), unemployment, relationship problems, and child protection problems.

“Unfortunately, I think that more alcohol abuse occurs in less privileged environments, although in certain privileged backgrounds alcohol abuse also exists, and, in my opinion, even more so…. There is also a problem of medication abuse among families who are a little less privileged; this is the feeling that I have”. GP14, Male (M), 62 y, Fr

Some GPs cited the facilitating role of the capitation payment system because it allows extended consultation times. However, it was also thought to improve access among illegal drug and alcohol abusers, increase the referral rate from addiction treatment centres, and increase the number of addicted patients.

“Expertise is increasing in the medical homes because it is well known that we work in multidisciplinary teams; there is more global care, better accessibility, [and] therefore, naturally, [one cares for] people who withdraw from therapy or leave the Alpha centre etc…. The [social] workers… will maybe say to themselves… that a medical home will be more suitable because [it is a] more integrated… type of care than service providers who are on their own”. GP17, M, 37 y, Fr

In contrast, the fee-for-service system and individual practices were mentioned as being less in favour of substance abuse management. Specifically, the GPs mentioned difficulties with regard to refusing prescriptions for hypnotics and tranquilisers.

“If a patient only comes for a prescription, which is common because it is fee-for-service, then it is sometimes difficult to say, ‘I am not going to prescribe [that drug]’. And then the patient stands there asking, ‘Will I have to pay, then?’ Yes, actually; but that does make it difficult, ethically speaking. In our community health centre, I simply tell them, ‘I’m sorry. We can’t do that’. We can easily refuse”. GP 9, F, 29 y, Dutch-speaking (Nl)

### Awareness factors: abuse management requires specific and nonspecific skills

GPs did not use peer-reviewed literature to support their practices. GPs, especially those in Flanders, mentioned the lack of guidelines regarding illicit drugs. GPs who supervised trainees in their practice more easily accessed such information.

“Mr. X reported that he occasionally uses cocaine. I think that is okay, but is it really okay? I would like to [review] the guideline; that [might] help me. [Then], if he comes back another time, I [would] know exactly what I should ask so that there is less guess work.... It does not have to be a novel or anything like that; something short… a consensus text, a guide… [that helps] you proceed with someone who reports [drug abuse]”. GP10, F, 43 y, Nl

The classifications of “misuse,” “addiction,” or “problematic use” were rarely known or used. The recommended maximum intake for alcoholic beverages by the World Health Organisation (WHO) was much better known than that for illicit drugs. Few GPs used screening tests for patients at risk, and some used the CAGE-test [28]. GPs did not consider systematic screening as part of their job, or they did not feel comfortable doing so. Only a few GPs mentioned the use of blood or urine tests.

“Yes. Imagine that you have come for a consultation for the first time, and you have [dysmenorrhea]. Should I ask whether you use drugs? Yes, I am somewhat reluctant to ask that of everyone as a standard question.... No, I do not do that. Maybe I should; I do not know…” GP 2, M, 51 y, Nl

A confident relationship based on strong interpersonal skills and a patient-centred approach seemed to predict the successful management of substance abuse. Although this competency was central, it seemed to be due to the personalities of the GPs in Wallonia; specific training to encourage this behaviour rarely occurred. In Flanders, GPs more often considered communication skills training as *conditio sine qua non* to manage these types of patients. These skills included motivational interviewing, cognitive behavioural therapy, and systems thinking. GPs described patient management as a package of tailored and flexible interventions, built around shared and realistic objectives, appropriate to the real world.

“That depends on the objectives that you set [for] yourself: Is it to reduce risk, or is it to put an end to substance abuse? It is important to define that at the beginning”. GP17, M, 37 y, Fr

“Yes, the first thing is to open it [up] for discussion. They have to feel that they can discuss anything here. And that it can be discussed in a non-normative manner, now and in the future.… I frame it as a dilemma. You have to be able to come up with your own agenda. And I should not be able to determine your agenda; that is one ideal”. GP 2, M, 51 y, Nl

Important differences in training among the GPs were reported. Those who were most involved in substance abuse management had undertaken Continuous Professional Development (CPD) or network collaborations concerning this topic. Young Flemish GPs trained in communication skills specifically expressed that this training was particularly helpful for substance abuse management.

“You see, I guess you could say that I was trained before the war. We read a bit of theory about substance [abuse], but we did not know anything about conversational techniques. And that is what you need: how you should address it with this person or that person”. GP 8, M, 59 y, Nl

The GPs discussed how their attitudes changed as a result of becoming more experienced. Starting from an idealistic or anxious point of view, feeling intrusive, with little life experience and only their education, some of them gradually moved toward a more pragmatic method of addressing abuse without antagonising the patient.

“For example, with a 60-year-old man with an alcohol problem… because of my age and the comfortable life that I lead, you almost feel guilty pointing fingers and saying, ‘You have a problem,’ you see. I cannot imagine it; I say that as well”. GP 1, F, 29 y, Nl

### Motivation factors: GPs’ personal representations influence management

Personal, familial, or professional experiences of substance abuse were mentioned as influencing GPs’ behaviours toward patients who exhibited these behaviours. Some GPs refused to treat these patients, whereas others cared for them with increased empathy and consideration. Personal histories, deep emotions, or emotional burdens influenced the GP’s choices with regard to addressing and managing patients who abuse substances. The balance between caring for one’s patients and caring for oneself seemed to directly affect GPs’ behaviour.

“I fell into a depression then as well. I learned a lot from it personally, but I certainly use it in my daily work as well. And I think that I can sense very quickly if someone is not feeling mentally up to par; I can recognise it quickly. The personal experience makes me more sensitive, I think. It certainly plays a role”. GP 3, F, 35 y, Nl

Because substance abuse management can be challenging and stressful, the GPs said they had to identify, assess, and control their own emotions when dealing with it. With experience, certain skills are developed and additional self-care strategies are adopted. Even when their motivations (i.e., attitudes, social influence, and self-efficacy) were highly positive, the fear of not being able to personally or emotionally cope might cause GPs to refrain from becoming involved.

“Is it artificial [to find a meaning in becoming involved with these patients]… to hold on and be happy in my job, even if it does not pay dividends? Or is it therapeutically useful? Well, that is my question”. GP 18, F, 32 y, Fr

The GPs’ perceived self-efficacy depended on positive physician-patient relationships, confidence in their own skills, and positive emotions. Time constraints and personal involvement were cited as important barriers for managing patients who required more time, especially when the chances for success are limited.

“No. That is not very easy for me. It is a sort of intimacy, like when you are talking about sex. Or… it has a normative character… or something like, “How dare you ask me that?”… I think that I project that onto the patient. I do not know whether the patient thinks that. Perhaps the patient thinks that it is a normal medical question”. GP 2, M, 51 y, Nl

When treating addicted patients face-to-face, two attitudes emerged: The GPs either considered substance abuse as a chronic disease (and therefore part of their routine clinical activities) or they expressed moral judgements about these patients, highlighting their faults and responsibilities with regard to clinical and social damages, and considering them untrustworthy. This second attitude reduced GPs’ willingness to manage substance abuse.

“I cannot stand drug addicts because they are liars, and I do not like liars; alcoholics are liars too, but the former are the worse, especially because of substitution therapies…. Drug addicts are utter and complete liars, and I believe that [caring for them is not my responsibility], it is the medical centres’”. GP 19, M, 58 y, Fr

GPs perceived a primary responsibility to manage substance abuse. Some GPs were strongly engaged in their coaching role, supporting patients with regard to accepting their responsibilities and providing strength to allow them to face life’s difficulties.

“When [I] treat an alcoholic who drinks and knocks someone over, I feel personally responsible…“. GP 11, M, 51 y, Fr

Some GPs also reported having a positive attitude with regard to this aspect of their job. In fact, many said that these responsibilities are what being a GP is all about.

“I always find it rewarding [to see] an alcoholic who is no longer dependent, who moves forward in life, who is more autonomous than before, who has a better quality of life in the broad sense. It is his life, it is his quality of life; but I think that if I, at any given moment, have been able to help him to reach this autonomy, well, that is good. […] I think that this is our role as doctors”. GP 13, M, 43 y, Fr

However, satisfaction with regard to dealing with addicted patients was rare. Substance abuse was described as a complex problem that requires long-term, staged follow-up assessments that proceed at the patient’s pace and are associated with many relapses without any outcome certainties. GPs often considered abstinence to be a long-term goal. Negative past experiences gave some GPs a feeling of impotence.

“There is weariness with regard to morally supporting people. We must accept (and the patients must also accept) that we may not be able to cure them, but that we are there to help them…. It is difficult to unceasingly return [to] a problem that one cannot solve; it is not very rewarding on a medical level; it is easier to cure people…” GP 12, F, 55 y, Fr

Some GPs considered the use of all illegal drugs as abuse, whereas others considered this use as abuse only when it affected the patient’s health or social life. Cannabis was often tolerated for recreational use and was considered common among young people. Alcohol was much more socially acceptable than other substances; moreover, alcoholism was easier to address than psychotropic drug abuse. Some GPs were more tolerant of psychotropic drug abuse (particularly among elderly people), whereas others considered it to be a growing problem, and some of them felt partly responsible for initiating psychotropic treatment among patients looking for help with critical life events.

### Intention state: GPs also proceed through a motivational process

The GPs were concerned about possible breakdowns in the therapeutic relationship; therefore, they often delayed the beginning of interventions until they perceived an opportunity to discuss the abuse with their patient. After the problem was broached, the patients were made aware that the GP’s door was open for additional dialogue. This behaviour was part of the contemplation stage.

During the preparation stage, the GPs sought opportunities to broach the subject with the patient, for example, when both parties had sufficient time and when the context was appropriate (i.e., not during mourning or immediately following job loss, divorce, and so on).

“Sometimes you ask about [the drug abuse], and you know that they are lying. Then you know, [now] is certainly not the time to go into any depth because the patient does not want to hear about it.... Then you just leave it alone for a while. In many cases, these are the types of patients who will be back. You just know that”. GP 1, F, 29 y, Nl

GPs relied on their clinical competence, and various reasons were used as opportunities to broach the problem of abuse, including repeated requests for sickness-absence certificates or drug prescriptions, physical stigmas and symptoms of acute trauma, social malfunctioning reported by either the patient (sometimes) or his or her relatives (more often), or simple intuition (i.e., a “gut feeling”).

“The family also puts pressure on us, especially the parents. There are parents who beg us to do something, really; and then we initially see the parents 3-4 times before seeing the child because the latter does not want to see us at all”. GP 14, M, 62 y, Fr

### Ability factors (and barriers): one cannot handle this alone

The GPs expressed their need to collaborate with other caregivers in multidisciplinary networks, primarily for psychological and social reasons. The opportunity to collaborate easily with other professionals was perceived as an advantage of multidisciplinary teams. Some GPs asked about a place for information and peer exchange for support in case of pitfalls and feelings of impotence. Others asked for financial and organisational incentives. In group practices, electronic medical records provide the opportunity to share information and alert GPs to possible drug abuse or patients at risk for aggression.

Referrals to psychiatrists or psychologists were difficult and often too expensive for most patients. The waiting list at specialised care centres was a major concern among the GPs.

“If you have a patient who has been addicted for a very long time, [then it is frustrating] once you have finally gotten him motivated to go into detox, and there is nothing available. People often drop out then, you know. They say, ‘I just don’t care anymore’ or ‘I don’t want it anymore’ or ‘I can solve my own problems’. And it is with precisely these people that you need to strike while the iron is hot, so to speak. This is the ideal time to admit someone. But then the moment is gone, and it is actually too late”. GP 7, M, 39 y, Nl

## Discussion

### Major results

A significant number of disparities exist among GPs with regard to their willingness to manage patients who abuse substances. The motivation to engage primarily depended on their personal attitudes, the available resources, and their training level. A striking feature of the analysis was that the vast majority of the statements ended up in the “attitude” portion of the I-Change Model. This portion of the model considered pros and cons that were crucial to determining the intentions and actions of substance abuse management, particularly with regard to its workability and manageability.

Another important finding was that the topic strongly affected all GPs, but these physicians were also highly concerned about protecting themselves as individuals and professionals because managing these patients requires time and energy. The fears of exhaustion and burnout were tangible and justified a demand for support, including exchange meetings or effective and accessible collaborations with specialised care centres.

Collaborative management was a prerequisite for GPs who sought support and a desire to share expertise, especially with regard to illegal drug users. Mental health care (at least its accessibility) was depicted as insufficient. Local collaboration within multidisciplinary practices might be an interesting solution.

### GPs’ representations of substance abuse

This study highlighted a great variety of behaviours linked to GPs’ personal histories and reflexivity with regard to treating patients with substance abuse, and these behaviours matched attitude and self-efficacy, as motivational factors [29]. Managing a patient with substance abuse is not a neutral care procedure for GPs because it elicits moral judgments. GPs’ perceptions of substance abuse were on a continuum from a chronic disease to a moral failing (for which the patient was completely responsible). The latter perception makes it difficult to trust the patient and engage in a constructive relationship to manage the problem.

Alcohol and psychotropic drugs are well known and more accepted by GPs; conversely, more reticence was expressed with regard to illegal drugs. It is not surprising that the opinions concerning these patients were more negative given the possible stereotypical views regarding substance use, as various authors have mentioned [9,13,14,30,31]. Goffman indicated that this stigmatisation leads to people spontaneously associating certain characteristics with substance abuse such as violence or untrustworthiness [32].

The various representations and social acceptability of different substances most likely depends partly on their legal status (e.g., alcohol and psychotropic drugs are more socially acceptable) and partly on their prevalence (e.g., cannabis is socially acceptable despite its illegality).

Older GPs with more professional experience tended to be more involved. Furthermore, perceptions of a clear role and defined limits with regard to their responsibilities protected GPs against feelings of frustration, disillusionment, and perceived impotence. However, attitudes can also be improved through communication skills training, peer exchange, and support. A strong difference existed between Dutch-speaking and French-speaking GPs concerning communication skills training.

The existing literature does not completely support the differences in prevalence between practice locations revealed in the interviews [33]. The link between precarious situations and substance abuse is also controversial [31,34]. The GPs tended to share opinions with laypeople regarding substance abuse rather than acknowledging that their personal attitudes can create biases for or against particular patients.

### Training

GPs’ attitudes concerning substance abuse, their perceptions of their role and their opinions concerning substance abuse as well as their lack of theoretical knowledge and training in this area are important determinants of their behaviours. The central effect of a physician’s personal qualities with regard to dealing with emotions, his or her personal life history/experience, self-care, and self-awareness in treating patients has been acknowledged previously. Bombeke et al. introduced the “doctor-as-a-person” model as a key determinant in the development of patient-centred behaviour among medical students [35]. Using this concept, they referred to the 5^th^ component of patient-centeredness as defined by Mead and Bower, which concerns a self-awareness of the influence of their personal qualities on the way they practice medicine [36].

Given the rich data that coincide with this “doctor-as-a-person” model, interventions that address self-reflection, coaching, or tutoring to improve self-care are advisable. Several participants suggested the need for this type of support, which can strengthen the ability factors of GPs. Specific skills are needed to maintain the delicate confidential doctor-patient relationship. Mutual respect is the appropriate attitude for helping these patients. Personal development, dealing with emotions and personal suffering, self-awareness, and self-care were submitted as key qualities that must be taught, guided, and fostered to maintain a patient-centred focus among GPs. Thus, tutorship and coaching are as important as theoretical and practical workshops in undergraduate education and continuous professional development programs, as various authors have mentioned over the past two decades [12,37-42]. Currently, these techniques are more commonly introduced in the medical curriculum of Flanders than that of Wallonia. This difference might explain why younger GPs in Flanders feel more comfortable managing patients with substance abuse.

### Collaboration

Addiction is a complex phenomenon that, according to WHO's definition of health, includes medical, social, and psychological aspects [43]. Collaborative care, which is an essential ability factor, is underdeveloped due to the limited accessibility of mental health care and social assistance facilities.

### Strengths and limitations

This qualitative exploratory study preliminarily analysed the determinants of GPs’ involvement in substance abuse management from the GPs’ points of view. This study cannot provide reliable information regarding influences at the macro-social level (e.g., the organisation of the healthcare system). Moreover, this study was conducted within a purposive sample with a limited number of participants.

This inductive phase should now be followed by a deductive phase. A quantitative survey will be conducted to measure the importance and prevalence of determinants of substance abuse management. The results might contribute to the implementation of policies that aim to support current practices. De Vries’ I-Change Model provided us with a complementary and continuous approach between the current qualitative portion and the upcoming quantitative portion of this study.

## Conclusions

This exploratory study highlighted major aspects of addiction management in the general practices of two Belgian provinces. The personal determinants of behaviour are most likely homogenous in culturally similar western nations.

Improving GP practice is often depicted as a matter of training or developing new tools to help physicians. Guidelines and implementation tools are of limited interest for those who do not favour personal involvement. Our study showed that GPs do not act as a homogeneous group. GP behaviours are strongly influenced by their opinions of substance abuse. Moral judgments and various fears were present in the therapeutic relationship. This point should be accounted for in the initial training of physicians. Support workshops and groups aiming to exchange best practices in a safe environment should also be considered for those who treat patients with substance abuse. This practice will help to break the isolation of GPs and reduce the risk of developing burnout, which is frequent among these professionals [44-46].

Improving substance abuse management in primary care is also a matter of policy as well as improving clinical competencies, as has been depicted for other mental health problems [47].

### Ethical approval

The Ethics committees of the Universities of Antwerp and Liege approved this study (Belgian Nr: 12/41/315 for Antwerp and B707201214939 for Liege).

## Competing interests

The authors declare that there are no competing interests.

## Authors’ contributions

All authors participated in the design of the study and construction of the interview guide.

FK, LS, ML and MV conducted the interviews; FK, LS and ML analysed the results with the help of LP and MV. FK drafted the manuscript. MV was the coordinator of the Up to Date research project, which includes this study. All authors read and approved the final manuscript.

## Pre-publication history

The pre-publication history for this paper can be accessed here:

http://www.biomedcentral.com/1471-2296/15/119/prepub

## Supplementary Material

Additional file 1 Interview guide. Click here for file
