# Supplementary material for: What factors determine Belgian general practitioners’ approaches to detecting and managing substance abuse? A qualitative study based on the I-Change Model
Source: BMC Fam Pract. 2014 Jun 14;15:119. doi: 10.1186/1471-2296-15-119 (PMC4064261; doi:10.1186/1471-2296-15-119)
Supplement: Additional file 1 — Interview guide. [file 1471-2296-15-119-S1.docx]

**Additional file 1: Interview guide**

| *GP definitions* | |
| --- | --- |
|  | First, the GPs’ personal definitions of substance abuse and the differences between use and abuse as well as problematic use and addiction were discussed. Then, the interview focused on a specific case from their practice. |
| *I-Change Model* | |
| Facilitators and barriers | Given this type of patient’s abuse, can you tell me some of the difficult management aspects you came across?  What allowed you to overcome obstacles and manage this case? Conversely, what were the compromises or concessions you made to continue with management?  Given the cases of abuse that you managed, what would help support you better? What factors contribute to making this job easier? |
| Skills and abilities | Do you feel comfortable in this area?  Where have you learned the most about this domain? What have you learned?  In your opinion, what competencies does a GP need to manage this problem well? To what extent do you think that you have these competencies? Have they improved with experience?  What is missing in your training? How could this absence be addressed? Do you have any suggestions? |
| Collaboration | Are there other laypeople/professionals concerned by this problem? Who? How so? |
| Context, Environment | Is it important for you to know patients’ employment statuses?  Are these elements addressed in relation to substance abuse? |
| Attitudes | In your opinion, what is the aim of substance abuse management? What type of results do you want to obtain?  Is the management of patients who engage in problematic substance use similar to that of patients with other health problems? |
| Norms and social influences | Did certain situations or people influence how you managed this problem?  How do patients influence this management? What is their role? |
| Self-Efficacy | Why do you decide to manage this problem? |
| *Miscellaneous* | |
| Physician as a person | To what extent does working on this problem satisfy you?  Do you think your personality or your life story affects your involvement in this management and its success or failure?  Did your behaviours or feelings concerning substance abuse develop throughout your career? |
| Professional status | Is the working context important? To what extent does it influence your management? |
| End of interview | Suppose you had the power and capacity to allow GPs to improve the management of patients with substance abuse. What would be your major ideas or measures to make this achievable? |
